# Proteomic analysis revealed the pharmacological mechanism of Xueshuantong injection in preventing early acute myocardial infarction injury

**DOI:** 10.3389/fphar.2022.1010079

**Published:** 2022-12-21

**Authors:** Aoao Wang, Ying Li, Ziyan Wang, Gaojie Xin, Yue You, Mingqian Sun, Lan Miao, Lei Li, Yinghong Pan, Jianxun Liu

**Affiliations:** ^1^ National Clinical Research Center for Cardiovascular Diseases of Traditional Chinese Medicine, Institute of Basic Medical Sciences of Xiyuan Hospital, China Academy of Chinese Medical Sciences, Beijing, China; ^2^ Institute of Crop Science, Chinese Academy of Agricultural Sciences, Beijing, China

**Keywords:** Xueshuantong injection, early acute myocardial infarction, proteomic analysis, protective mechanism, molecular docking

## Abstract

**Background:** Acute myocardial infarction (AMI) is a common and life-threatening cardiovascular disease. However, there is a lack of pathology and drug studies on AMI within 20 min. Xueshuantong injection (XST) is mainly composed of *Panax notoginseng* saponins, which can dilate blood vessels and improve blood circulation, and is clinically used in the treatment of cardiovascular and cerebrovascular diseases.

**Purpose:** The study aimed to investigate the protective mechanism of Xueshuantong injection against acute myocardial infarction within 20 min in rats by proteomic methods and molecular docking.

**Method:** The male Sprague–Dawley rat acute myocardial infarction model was established by LAD ligation, and Xueshuantong injection (38 mg/kg) was injected into the caudal vein 15 min before surgery. Cardiac function evaluation, morphological observation, label-free quantitative proteomics, Western blotting analysis, molecular docking, and affinity measurement were applied in this study.

**Results:** In a span of 20 min after acute myocardial infarction, the model group showed significant cardiac function impairment. Xueshuantong injection can significantly improve cardiac function and prevent pathological injury of myocardial tissue. A total of 117 vital differentially expressed proteins were identified by proteomic analysis, including 80 differentially expressed proteins (DEPs) in the sham group compared with model rats (Sham: model) and 43 DEPs in model rats compared with the Xueshuantong injection group (Model: XST). The treatment of Xueshuantong injection mainly involves “poly(A) RNA binding” and “cadherin binding involved in cell–cell adhesion.” The differentially expressed levels of the pathways related to proteins Echdc2, Gcdh, Dlst, and Nampt, as well as 14-3-3 family proteins Ywhaz and Ywhab, could be quantitatively confirmed by WB. Molecular docking analysis and SPR analysis revealed that Ywhaz has a generally stable binding with five Xueshuantong injection components.

**Conclusion:** Xueshuantong injection (XST) could protect rat myocardial function injury against AMI in 20 min. Echdc2, Ywhaz, Gcdh, Ywhab, Nampt, and Dlst play an essential role in this protective effect. In particular, Ywhaz might be the core target of Xueshuantong injection when treating acute myocardial infarction in the early stage. This study promoted the understanding of the protective mechanism of Xueshuantong injection in 20 min injury of acute myocardial infarction and contributed to the identification of possible targets of Xueshuantong injection.

## 1 Introduction

Acute myocardial infarction (AMI) remains one of the most common causes of death globally (Judit et al., 2021), which results in an insufficient supply of blood and oxygen and may affect myocardial function at rest and during stress (Michelsen et al., 2018). Diagnostics of the initial stage of AMI have important implications in not only clinical settings for the survival and treatment of patients but also in assisting legal cases and counseling for dependents in medico-legal situations (Aljakna Khan et al., 2021). However, the physiological change in this period is still unclear. The hyperacute phase (reversible phase) of myocardial infarction refers to 30 min after myocardial infarction (Zipes et al., 2007). Generally considering, this short duration is a problematic diagnosis with reversible myocardial injury, so the related pathophysiological mechanism is seldom studied. However, the hyperacute phase of myocardial infarction can easily cause ventricular fibrillation, which increases the risk of sudden death in patients with myocardial infarction and causes severe adverse consequences (Zipes et al., 2007). This pathological process can be defined at the molecular level as an assembly of hundreds of intracellular and extracellular proteins that collectively alter biological processes and produce pathological damage (Du et al., 2019; Aljakna Khan et al., 2021). With the development of proteomic technology, these changes can be qualitative and quantitative and then be used to discover drug targets and explain the mechanism of drug action (Herrington et al., 2018; Du et al., 2019; Kalyanasundaram et al., 2021; Cui et al., 2022).

Xueshuantong (XST) is developed from traditional Chinese medicine (TCM) to prevent and treat cerebral myocardial infarction and significantly contains *P. notoginseng* saponins (PNS). Studies have shown that XST has effects on anti-myocardial infarction (Liu et al., 2021), antithrombotic (Han et al., 2019), anti-cerebral ischemia/reperfusion injury (Zhang et al., 2021), antioxidant (Peiran et al., 2017), and amelioration of microcirculation disorders (Han et al., 2017). The clinical use of XST for AMI (Fu et al., 2009), coronary heart disease, angina pectoris (Long et al., 2020), cerebral infarction (Zeng et al., 2021), microcirculation disorders, and other ischemic cardiovascular and cerebrovascular diseases has been reported historically. However, the related pathological mechanism of anti-hyperacute myocardial infarction and the drug action mechanism are not precise.

**FIGURE 1:**
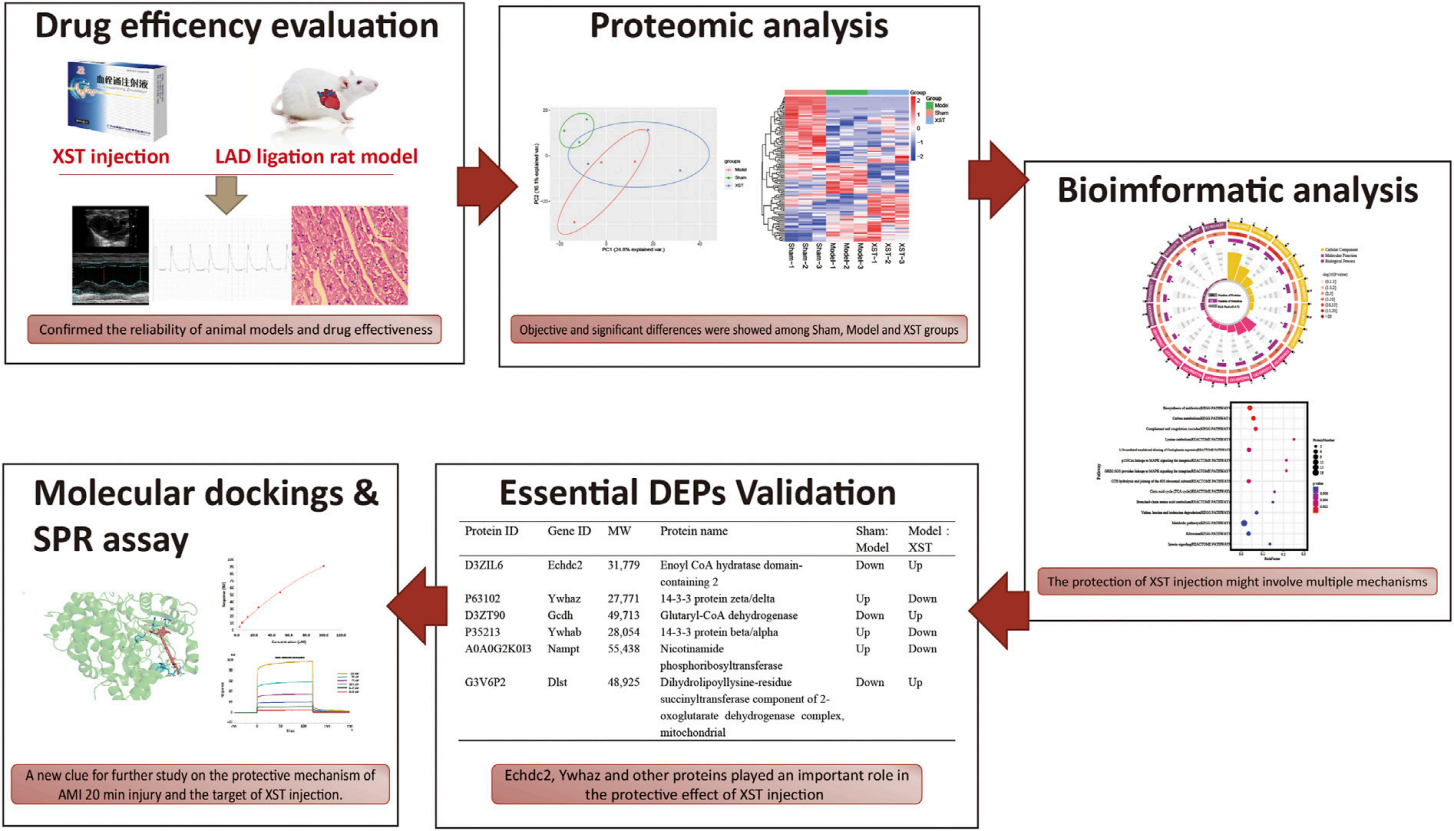
Potential mechanism of XST in the ameliorative effect of AMI is to work on fatty acid metabolism and platelet procoagulant function, which is closely related to the binding of the major active component ginsenoside Rb1 and the potential therapeutic target Ywhaz.

This study was designed to investigate the efficacy and mechanism of XST in alleviating hyperacute myocardial infarction injury in rats. In addition, differential proteomics was used to explore the target protein of XST, and combined with bioinformatics analysis, the possible biological mechanism of its therapeutic effect was discussed (Figure 1).

## 2 Materials and methods

### 2.1 Chemicals and materials

Xueshuantong, with the Chinese NMPA drug ratification number GuoYaoZhunZi-Z45021769, has been manufactured as a sterile and non-pyrogenic liquid for intravenous administration. Each milliliter of XST was prepared from 35 mg of Sanqi [*P. notoginseng* (Burk.) F. H. Chen]. XST used in this study was obtained from the Guangxi Wuzhou Pharmaceutical Group with the lot No. 200901 (Wuzhou, Guangxi Zhuang Autonomous Region, China). Diltiazem hydrochloride for injection was purchased from Tianjin Tanabe Seiyaku Co., Ltd. with the lot No. F030. RIPA lysis buffer, dithiothreitol (DTT), iodoacetamide (IAA), urea, and NH_4_HCO_3_ were purchased from Beyotime. Recombinant Mouse 14-3-3 protein zeta/delta (Ywhaz) was purchased fromCUSABIO (https://www.cusabio.com/) with lot No.YA0403a1g5. Ginsenoside Rg1 (ChromaDex Inc., ASB-00007221-100), ginsenoside Rb1 (National institute for food and drug control, LA38-E2PK), notoginsenoside R1 (Chengdu Herbpurity Co., Ltd, RDD-S00202010012), ginsenoside Re (National institute for food and drug control, 62JZ-1FP3) and ginsenoside Rd (Shanghai yuanye, Bio-Technology Co., Ltd, Z09A9X67397) were dissolved in DMSO.

### 2.2 HPLC-Q-TOF-MS analysis condition

A proper amount of XST was accurately absorbed and diluted with 20% acetonitrile into a solution containing about 5 μg/ml of total saponins. The collection mode was positive ion, capillary voltage was 3,500 eV, atomization temperature was 350°C, dry gas was 10.0 L/min, and atomization gas was 206.85 kPa. The scanning range of ms was 80–1,000 m/z, and the data storage mode was centroid. The ion source was a dual-ESI spray, and the data were collected by mass spectrometry using Hexakis (1H, 1H, 3H tetrafluoropropoxy) phosphazine and 7H-purine, respectively. The real-time correction was performed for 922.009 80 and 121.050 9 m/z. The HPLC system was Agilent 1209. An RP column (Atlantis, T3, 150 mm × 2.1 mm, 3 μm, Waters, IRELAND) was used for liquid chromatography. The mobile phase consisted of 0.1% formic acid aqueous solution (A) and 0.1% formic acid acetonitrile solution (B). The gradient elution was 0–30 min, 12%–30% B, stopped at 30 min, and balanced at 12% B for 5 min. The flow rate was 0.25 ml/min, the column temperature was 35°C, and the injection volume was 5 μl.

### 2.3 Animal grouping, treatment, and modeling

A total of 40 male Sprague–Dawley (SD) rats weighing 220–250 g were purchased from the Vital River Laboratory Animal Center (Beijing, China). All animal experimental protocols were approved by the Ethics Committee of Xiyuan Hospital (2022XLC041). The animals were maintained under conventional conditions in our animal facility with a 12-h light and dark cycle with access to regular food and tap water. Then, the rats were randomly divided into four groups (*n* = 10 per group), namely, the sham group, model group, diltiazem group (1.0 mg/kg), and XST group (38 mg/kg), and were treated by a tail vein injection. Operation was performed 15 min after administration. The AMI model was established by ligation of the left anterior descending (LAD) branch. AMI was confirmed by elevation of the ST segment on an electrocardiogram and bulging of the relevant segment of the left ventricle (LV). In the sham group, the suture was removed without tying, and no infarction was generated.

### 2.4 Test of hemodynamics of the artery and left ventricle (LV)

The heart function was tested using a biofunction experiment system MP-150 multi-guide physiological recorder *via* a DA 100C amplifier (BIOPAC Systems Inc., CA, United States), which was connected to a cannulation inserted into the left ventricle through the right carotid artery. The heart rate (HR), left ventricular systolic pressure (LVSP), left ventricular diastolic pressure (LVDP), left ventricular end-diastolic pressure (LVEDP), left ventricular maximum upstroke velocity (+*dp*/*dt*_max_), and left ventricular maximum descent velocity (−*dp*/*dt*_max_) were evaluated after 15 min of operation.

### 2.5 Electrocardiogram (ECG) and echocardiography

After 18 min of operation, cardiac function was assessed within 2 min. The surface ECG in standard lead II was recorded using a Softron ECG processor system (Softron, Tokyo, Japan). Then, the echocardiography ultrasound transmission gel was applied to the chest, and echocardiography (M-mode and B-mode imaging) was performed in a VisualSonics Vevo 2100 high-resolution ultrasound imaging system for small animals (FUJIFILM VisualSonics, Inc., Toronto, Canada). The LV fractional shortening (FS), ejection fraction (EF), cardiac output (CO), and stroke volume (SV) were measured in each rat in a blinded manner. All values were averaged using three to five cardiac cycles per rat.

### 2.6 Cardiac histopathological examination

Rat hearts were harvested after 1% pentobarbital sodium overdose *via* intraperitoneal injection and washed in physiological saline. The left ventricular region of each group was fixed in 4% paraformaldehyde for 24 h. After fixation, it was embedded with paraffin and stained with hematoxylin and eosin (HE). The pathological changes of myocardial injury in each group of rats were observed using an optical microscope (Carl Zeiss Microscopy GmbH, Göettingen, Germany).

### 2.7 Protein extraction and digestion

The myocardium in the marginal zone of the infarct region in model animals was harvested. The left ventricular myocardial below ligation bit in sham animals was also dissected. The samples were then immediately frozen in liquid nitrogen and stored at −80°C. Frozen tissue from three animals of each group (from −80°C) was weighed and ground with the tissue extraction medium [RIPA lysis buffer, 1 mM PMSF, and protease inhibitor cocktail (1:50)] (1 mg: 10 μ) using a freezing grinder (Shanghai Jingxin Industrial Development Co., LTD., China). The samples were centrifuged at 15,000 g at 4°C for 20 min to remove the insoluble material. After centrifugation, the protein concentration of each sample was quantitated using a BCA assay and used for the following processes. After adding the sample to a 10-kDa filter and washing it with 8 M urea three times, the total protein extracted from each sample was chemically reduced for 30 min at 37°C by adding DTT to a concentration of 10 mM. Next, the protein was washed with 8 M urea three times and carboxyamidomethylated in 20 mM iodoacetamide for 30 min at 37°C in the dark. Then, the protein was washed with 8 M urea three times and 50 mM ammonium bicarbonate three times. Trypsin (Promega) was added to a final substrate: enzyme ratio of 50:1 (w/w). The trypsin digest was incubated at 37°C for 12 h. After digestion, the peptide mixture was acidified by adding 10 μ of 0.1% formic acid for further MS analysis.

### 2.8 LC-MS/MS analysis

The peptides for DDA and PRM analyses were analyzed by Easy-nLC 1,000 nL liquid chromatography–tandem mass spectrometry using a Q-Exactive plus mass spectrometer (Thermo, United States). The binary solvent system was made up of 99.9% water and 0.1% formic acid (solvent A) and 99% acetonitrile and 0.1% formic acid (solvent B). The mobile phase gradient is as follows: 0–25 min, 5%–9%B; 25–65 min, 9%–23%B; 65–75 min, 23%–32%B; 75–76 min, 32%–95%B; and 76–90 min, 95%–95%B. Flow rate: 420 nl/min. Eluted peptides were analyzed by DDA and PRM methods. To correct the instrumental error, each experimental sample was analyzed in triplicate.

Primary mass spectrometry observation was detected at 70,000 resolution and was collected from the mass analyzer using the full ion scan mode over the m/z range 400–1600. The automatic gain control was set to 3E6, and the injection time was set to 50 ms. The resolution of the secondary mass spectrometry was set to 17,500, the automatic gain control value was set to 1E5, and the injection time was set to 45 ms. The collision energy was set to 27 NCE.

### 2.9 Protein identification and quantitative analysis

After obtaining the original data, the protein types were searched by Proteome Discoverer software, and the rat protein sequences were searched using the protein information in the UniProt database (http://www.uniprot.org/). The parameters were set as follows: peptide confidence: high, the maximum number of missed protein sites: 2, peptide detection length: 6–144 amino acids, mass deviation of parent ion: ±10 PPM, mass deviation of fragment ion: 0.02 Dalton, fixed modification: cysteine (Cys), and variable modification: methionine (Met) oxidation. A maximum false positive rate of 1% was allowed.

MaxQuant software was used to quantify the protein, and the protein sequence was obtained from the UniProt database. The parameters were set as follows: maximum of two missing protein sites, fixed modification: cysteine and iodide acetamidation, variable modification: methionine oxidation and N-acetylation, mass deviation of fragment ions: 0.02 Dalton, and minimum detection value of peptide: seven amino acids. A maximum false positive rate of 1% was allowed.

### 2.10 Bioinformatics analysis

After obtaining the quantitative information, the differential proteins (FC > 1.2 or < .83, *p* < .05) can be analyzed by online and offline bioinformatics software applications to find the related biological pathways and network interaction information. The Gene Ontology database (http://geneontology.org/) and UniProt database (https://www.uniprot.org/) were used to analyze protein classification, functions, cellular localization, and the biological pathways. The DAVID (https://david.ncifcrf.gov) database, KEGG (www.genome.jp/kegg/) database, and STRING (http://www.string-db.org/) database were used to enrich the biological functions of proteins.

### 2.11 Validation of differentially expressed proteins by Western blot analysis

The left ventricular protein was extracted with RIPA lysate containing protease inhibitors, and the protein concentration was determined by the BCA method. SDS-PAGE was performed, electrophoresed to nitrocellulose membrane, and blocked with 5% skim milk for 1 h. Next, antibodies and dilutions were as follows: Gcdh (Proteintech, 14930-1-AP, rabbit), Ywhaz (ABclonal, A13370, rabbit), Ywhab (ABclonal, A1023, rabbit), Nampt (Abcam, ab236874, rabbit), Echdc2 (ABclonal, A14591, rabbit), and Dlst (ABclonal, A13297, rabbit). The fluorescently labeled secondary antibodies were then conjugated by incubation. The blotted proteins were quantified on the Odyssey Infrared Imaging System. The ratio of the target protein to the internal reference protein *β*-actin reflects the relative expression of the protein.

### 2.12 Molecular docking

Probable binding structures of XST ingredients and validified DEPs were obtained using a molecular docking study using the docking program AutoDock 4.2. The structures of the five major compounds of XST were obtained from the PubChem database (https://pubchem.ncbi.nlm.nih.gov), whereas the X–ray crystal structure of six core validified DEPs was obtained from the RCSB Protein Data Bank (http://www.rcsb.org). Ligand compounds experienced the addition of non-polar hydrogen, calculated the Gasteiger charge, and set all the flexible bonds of small-molecule ligands to be rotatable. The receptor protein was set to rigid docking, the genetic algorithm was selected, and the maximum number of evals was set as the medium. The docking results were obtained by running autogrid4 and autodock4, by which the binding energies were revealed, and PyMOL 4.5 software was used to visualize the results from the molecular docking analyses to obtain 3D images.

### 2.13 SPR assay

The surface plasmon resonance (SPR) technology is a powerful tool for real-time monitoring of the interaction of binding kinetics and affinity. The SPR experiment was performed using the Biacore T200 SPR instrument (Biacore, GE Healthcare Life Sciences, Uppsala, Sweden) and sensor chip CM5 (carboxymethylated dextran surface, BR-1005-30, Cytiva). First, the protein Ywhaz was diluted to 30 μg/ml in immobilization buffer (10 mM NaAC pH = 4.0) and was immobilized *via* amine groups in the flow cell. The LWM was dissolved in DMSO (V900090, Sigma-Aldrich) and was diluted with 5% DMSO PBS-EP buffer solution successively to 500, 100, 50, 25, 12.5, 6.25, 3.125, and 50 μM. The sample was injected into the flow cell at a flow rate of 30 μl/min. The protein binding time was set to 120 s, and the dissociation time was 600 s. The chip was regenerated with glycine-HCl (pH 4.5, 10 mM). The data were retrieved and analyzed by Biacore T200 Evaluation Software, assuming the steady-state affinity analysis model.

### 2.14 Statistics analysis

Data were expressed as the mean ± standard deviation. The comparison of the results from various treatments was performed by SPSS 26.0 software with one-way ANOVA with LSD comparison analysis. *p* < .05 was considered statistically significant, and *p* < .01 was considered highly significant.

## 3 Results

### 3.1 Chemical component quality of Xueshuantong injection

**FIGURE 2:**
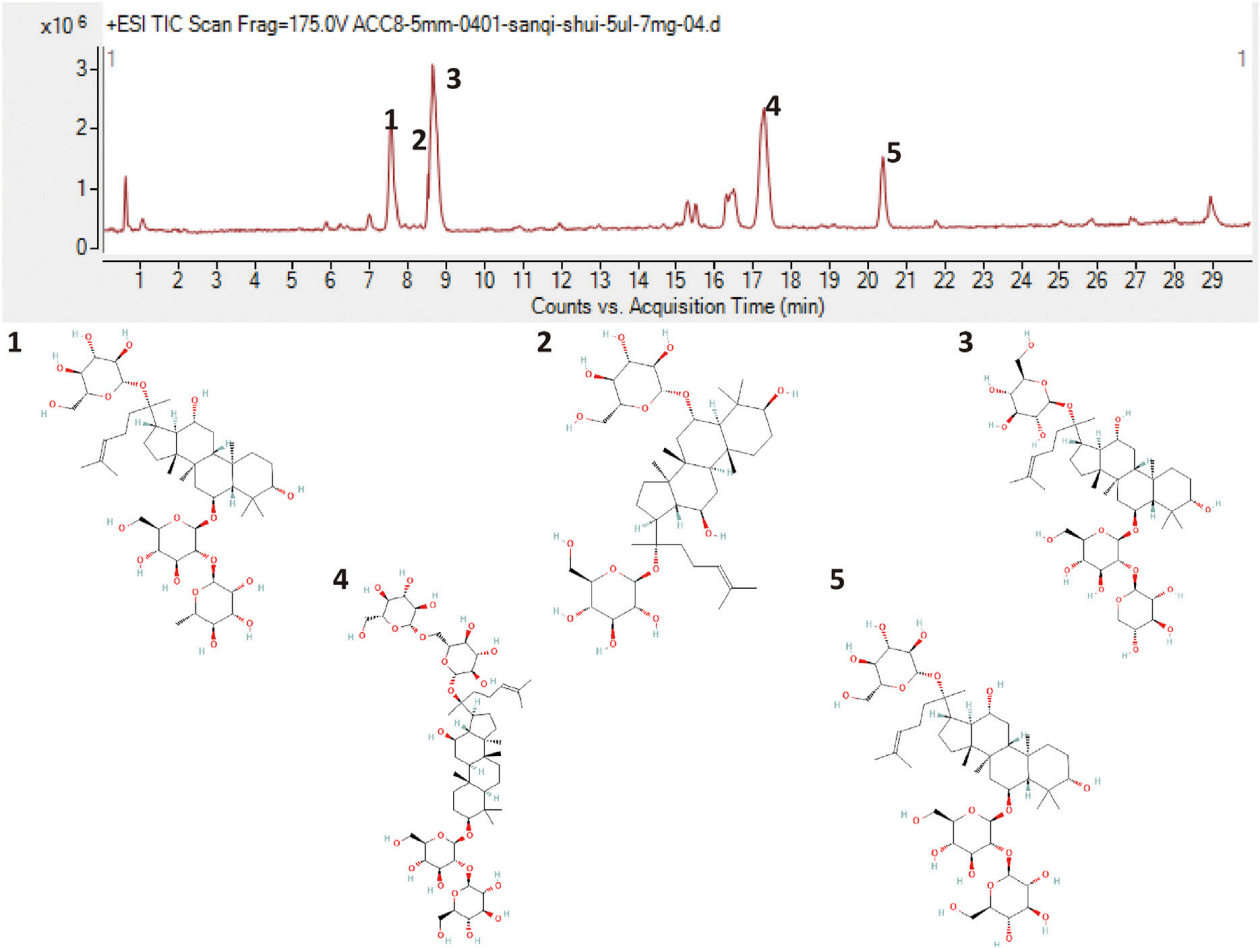
Liquid chromatogram of the XST sample and the chemical structures of five major ginsenosides. 1: Notoginsenoside R1. 2: Ginsenoside Rg1. 3: Ginsenoside Re. 4: Ginsenoside Rb1. 5: Ginsenoside Rd.

XST was purchased from Guangxi Wuzhou Pharmaceutical Group with lot No. 200901 (Wuzhou, Guangxi Zhuang Autonomous Region, China). To qualify the components of Xueshuantong injection, it was analyzed by HPLC-Q-TOF-MS and compared with previous qualification research, as shown in Figure 2. Five major compounds were identified from XST, containing saponins ginsenoside Rg1 (43.5%), ginsenoside Rb1 (27.8%), notoginsenoside R1 (12.6%), ginsenoside Re (5.6%), and ginsenoside Rd (2.3%), and these ginsenosides make up 91.8% (reference with Liu et al., 2021; Yao et al., 2017).

### 3.2 Xueshuantong injection promotes cardiac function against AMI in 20 min

**FIGURE 3:**
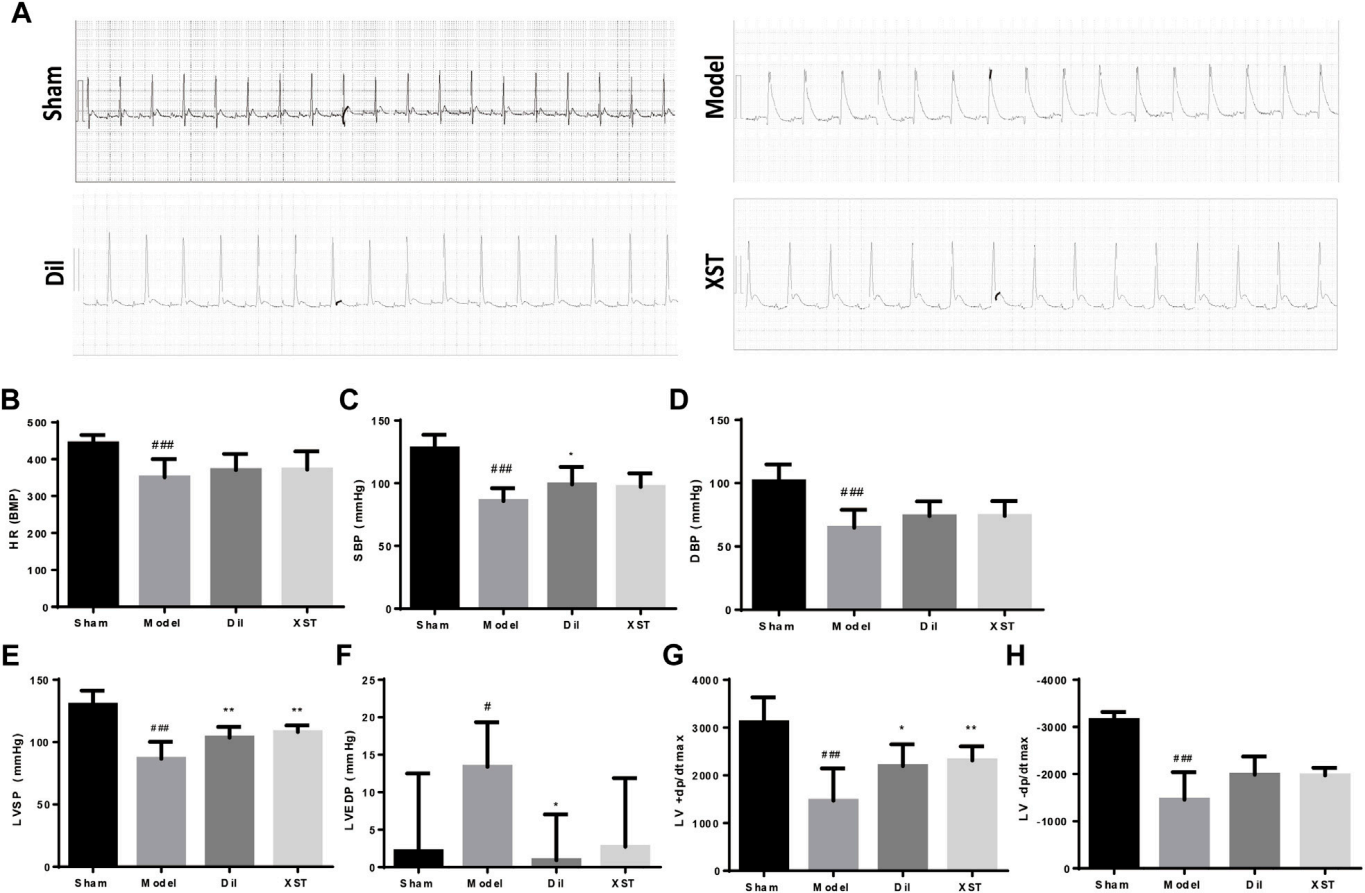
Effects of XST on ECG and hemodynamics in rats exposed to early AMI injury. **(A)** ECG of each group. The values of HR **(B)**, SBP **(C)**, DBP **(D)**, LVSP **(E)**, LVEDP **(F)**, +*dp*/*dt*_max_ **(G)**, and −*dp*/*dt*_max_ **(H)** were presented in various groups, *n* = 6–9. Data are presented as mean ± SD. ^#^ *p* < .05 vs. the sham group and *p < .05 vs. the model group.

As shown in representative ECG images (Figure 3A), the S-T segment was significantly elevated in the model group within 20 min of ischemia, whereas prophylactic treatment with Dil and XST significantly attenuated ischemic-induced change in ECG.

As demonstrated in Figures 3B–H, 20 min of ischemia caused a noticeable decline in HR, SBP, DBP, LVSP, +*dp*/*dt*_max_, and −*dp*/*dt*_max_ (^#^ *p* < .05 vs. Sham) and an increase in LVEDP, indicating impaired cardiac function. However, this impairment was generally alleviated by prophylactic treatment with Dil and XST. Notably, +*dp*/*dt*_max_ and LVSP were ameliorated by both Dil and XST. (*p < .05 and **p < .01 vs. the model group).

**FIGURE 4:**
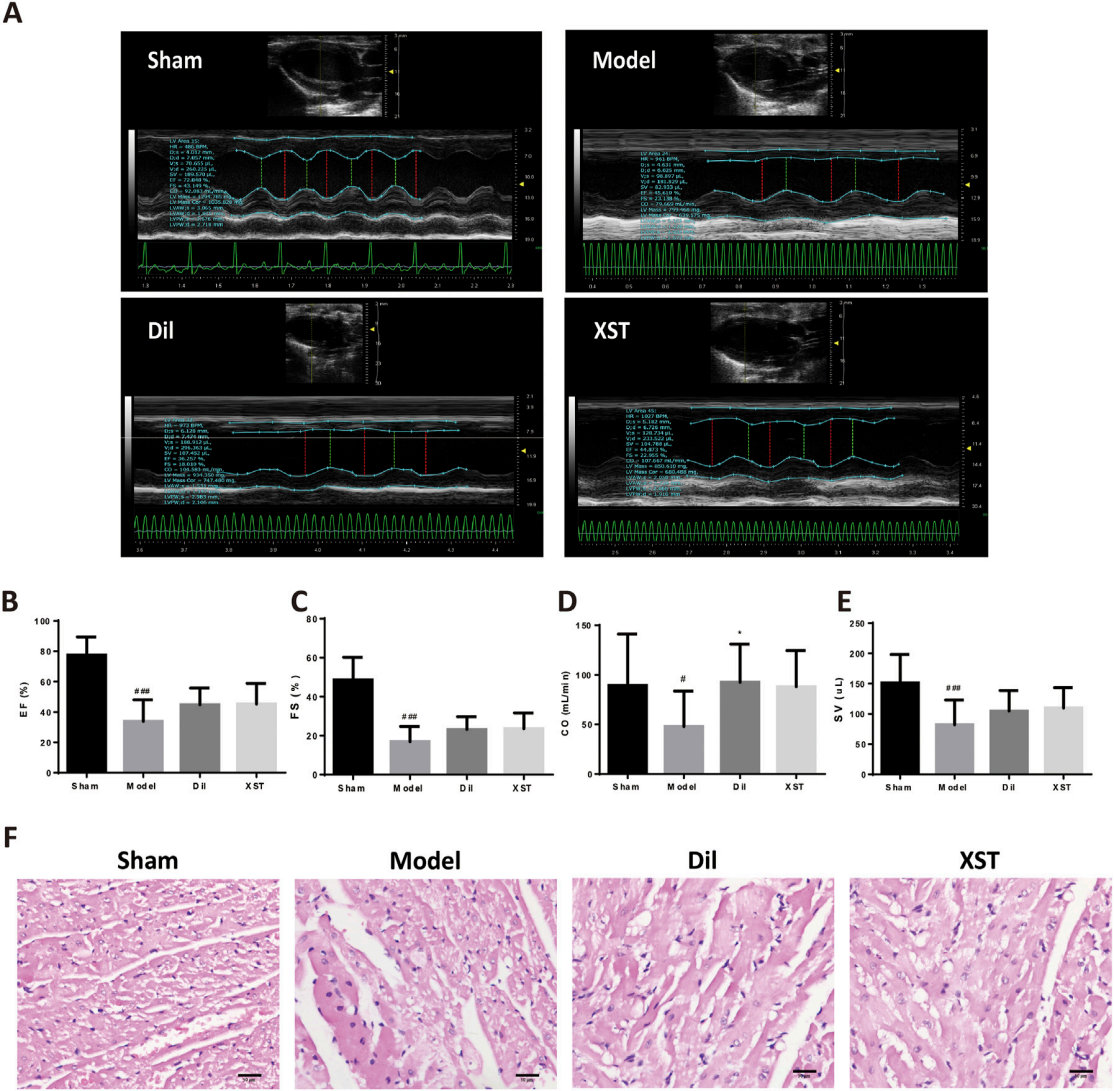
Effect of XST on cardiac function and myocardial histology in the AMI rat in 20 min. **(A)** Representative echocardiograph from the various groups. The values of EF **(B)**, FS **(C)**, CO **(D)**, and SV **(E)** in various groups, *n* = 8–13. **(F)** Representative HE-stained images in the sham (f1), model (f2), Dil (f3), and XST (f4) groups. The panel was captured at ×40 magnification of the objective. Bar = 50 μm.

The values of EF, FS, and SV were significantly lower in the model group than those in the sham group (^#^ *p* < .05 vs. the sham group), and CO had a decreasing trend, indicating an impairment in cardiac pumping function (Figures 4A–E). However, we found that the prophylactic treatment of Dil and XST significantly improved the cardiac function of AMI rats.

### 3.3 Xueshuantong injection retains rat myocardial morphology against AMI in 20 min

Figure 4F shows the myocardial morphology stained by HE in the sham group (f1), model group (f2), Dil group (f3), and XST group (f4). Compared with the sham group, ischemia for 20 min caused obvious morphological damage, such as myocardial space widening, cell edema, scattered nuclear deviation of left ventricular myocardial tissue, and cell vacuole into signet ring cells. Prophylactic treatment of Dil and XST can ameliorate myocardial structural damage after 20 min of ischemia.

### 3.4 Label-free quantitative proteomics analysis of myocardial tissue in the left ventricle from AMI- and XST-treated rats

**FIGURE 5:**
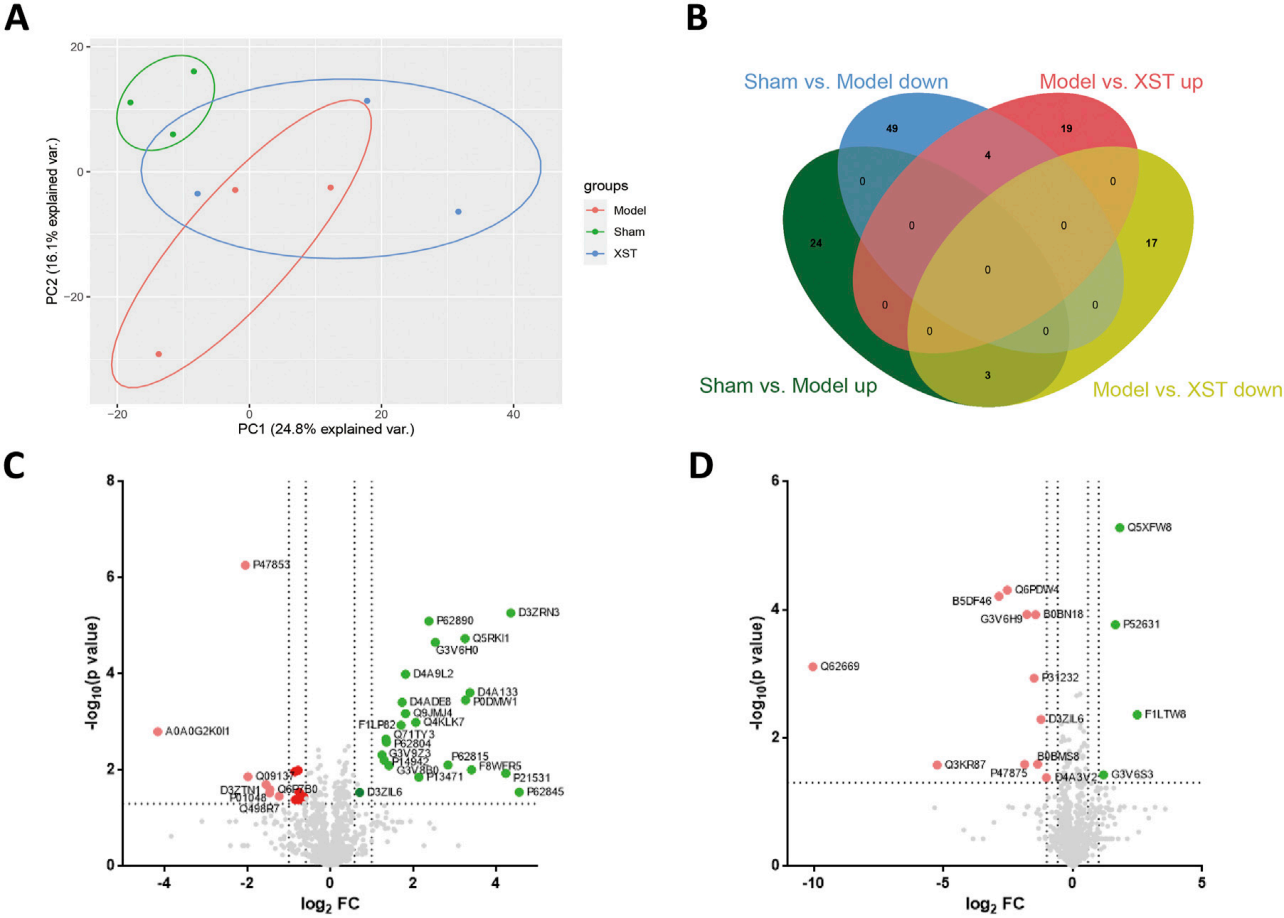
Differentially expressed proteins (DEPs) detected by label-free proteomics analysis in the left ventricle between Sham: model and Model: XST. **(A)** Principal component analysis (PCA) of all detected proteins of the three groups: the sham group, model group, and XST group. **(B)** Venn diagram showing the overlap of all DEPs. **(C)** Volcano plot of DEPs of Sham: model. **(D)** Volcano plot of DEPs of Model: XST. Red dots represent upregulated DEPs, green dots represent downregulated DEPs, and gray dots represent unchanged proteins.

To further investigate the potential mechanism of cardio-protective effects triggered by XST, the label-free quantitation method was performed to assess and quantify the overall proteomic concentration of the sham group, model group, and XST treatment group. The mass spectrometry proteomics data have been deposited to the ProteomeXchange Consortium (http://proteomecentral.proteomexchange.org) *via* the iProX partner repository (Ma et al., 2019) with the dataset identifier PXD035370. A total of 1,509 proteins and 6,858 peptides were successfully identified. The principal component analysis (PCA) model was constructed to visualize the classification pattern. As shown in Figure 5A, the clear separation between the sham group and model group was observed in the score plot established based on the data obtained from the LFQ intensity of each detected protein. After treatment with XST, the point from the XST group showed a tendency to separate from the model rats to the sham group and had an intersection with the aforementioned two groups, respectively, suggesting treatment with XST could partly recover the protein expression changes to the sham group and exert the cardioprotective effect to the AMI in 20 min. At the same time, 117 DEPs were detected among the three groups and displayed in a Venn diagram (FC > 1.2 or < .83, *p* < .05) (Figure 5B). Among these DEPs, 80 DEPs were explicated in Sham: model, 53 of which were downregulated and 27 were upregulated. The volcano plot of Sham: model is shown in Figure 5C. The volcano plot of Model: XST highlighted in Figure 5D shows that 43 proteins were explicated in Model: XST, of which 20 were downregulated and 23 were upregulated.

### 3.5 Bioinformatics analysis of differentially expressed proteins

**FIGURE 6:**
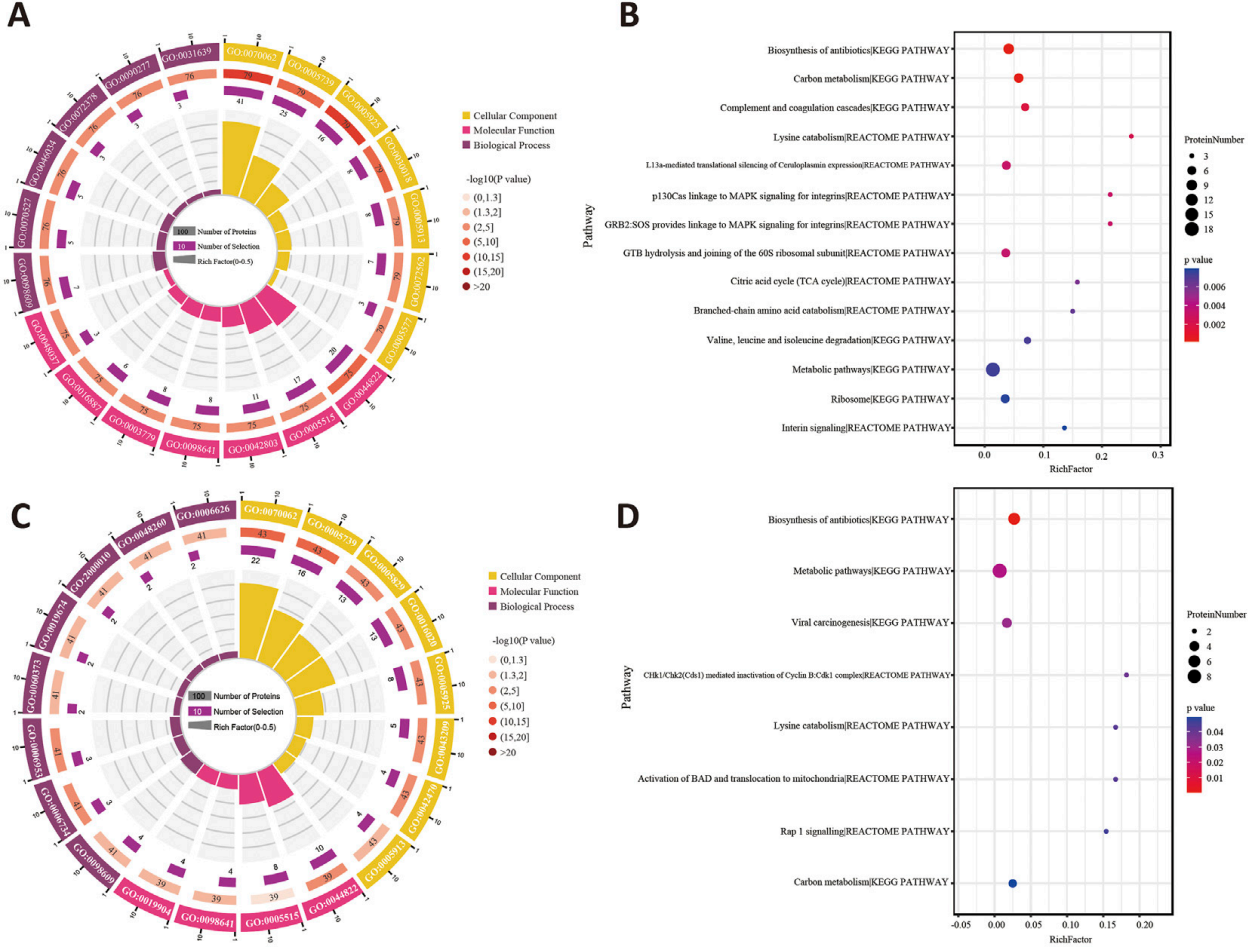
**(A)** Enriched GO terms for the DEPs of Sham: Model using the biological process, cellular component, and molecular function. **(B)** Statistics of pathway enrichment for the DEPs between the sham group and model group. **(C)** GO annotation analysis of Model: XST. **(D)** Classification of the enriched KEGG pathways and Reactome pathways for the DEPs between the model and XST groups.

The GO terms between the sham group and model group that showed remarkable enrichment are shown in Figure 6A. Biological process (BP) analysis showed that the majority of the differentially expressed proteins (DEPs) identified in Sham: Model was classified as platelet aggregation (GO:0070527) and the ATP metabolic process (GO:0046034). Moreover, molecular function (MF) analysis showed that the identified DEPs were predominantly involved in poly(A) RNA binding (GO:0046034). Concerning cellular component (CC) terms, most of the identified DEPs were associated with the extracellular exosome (GO:0046034) and focal adhesion (GO:0046034). Then, the pathway enrichment was statistically analyzed. As shown in Figure 6B, the KEGG pathway and Reactome annotation displayed that the main signaling pathways undergoing modulation were those related to the “biosynthesis of antibiotics” and “carbon metabolism.”

On the other hand, the BP analysis between the model group and XST group demonstrated that the NADH metabolic process (GO:0046034) and acute-phase response (GO:0046034) were changed the most. In addition, major identified DEPs were associated with poly(A) RNA binding (GO:0046034) in molecular function and concentrated in the extracellular exosome (GO:0070062) and mitochondrion (GO:0070062) (Figure 6C). Statistical analysis of pathway enrichment showed that the most significant changes occurred in the “biosynthesis of antibiotics” (Figure 6D).

**FIGURE 7:**
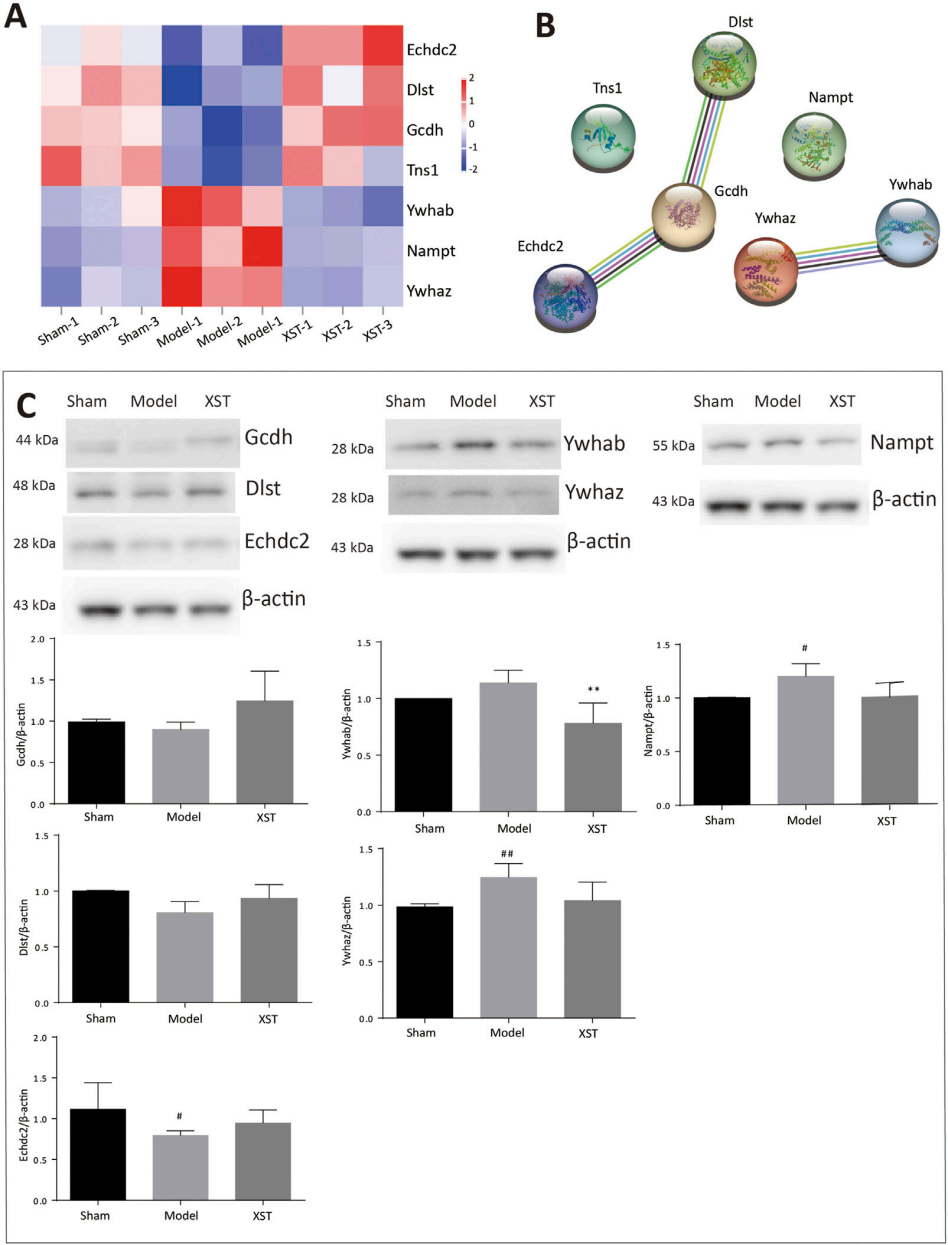
**(A)** Hierarchical analysis of seven shared DEPs in Sham: Model and Model: XST. Blue indicates a negative correlation, and red indicates a positive correlation. The darker the color, the more substantial the alteration is. **(B)** Protein–protein interaction network. **(C)** Effect of XST on AMI-induced alteration in six critical DEPs by Western blotting validation. *n* = 3. ^#^ *p* < .05, ^##^ *p* < .01 vs. the sham group, and **p < .01 vs. the model group.

To better visualize the differences in protein abundance among these three groups, hierarchical cluster analysis of the DEPs is displayed in Figure 7A. Among these, seven of the shared DEPs in the Sham: Model and Model: XST comparisons demonstrated that these proteins might be involved in XST-mediated cardioprotective effects against myocardial infarction (Table 1). The protein–protein interaction network of these DEPs was screened and input into STRING 11.5 to determine interactions among forecast functions of the DEPs (Figure 7B).

**TABLE 1:** Critically shared DEPs in the Sham: model and Model: XST comparisons.

Protein ID	Gene ID	MW	Protein name	Fold change (Sham: model)	Fold change (Model: XST)
D3ZIL6	*Echdc2*	31,779	Enoyl CoA hydratase domain-containing protein 2	.71	−1.22
P63102	*Ywhaz*	27,771	14-3-3 Protein zeta/delta	−.30	.30
D3ZT90	*Gcdh*	49,713	Glutaryl-CoA dehydrogenase	.34	−.44
P35213	*Ywhab*	28,054	14-3-3 Protein beta/alpha	−.29	.42
A0A0G2K0I3	*Nampt*	55,438	Nicotinamide phosphoribosyltransferase	−.46	.44
G3V6P2	*Dlst*	48,925	Dihydrolipoyllysine-residue succinyltransferase component of the 2-oxoglutarate dehydrogenase complex, mitochondrial	.30	−.33
F1LN42	*Tns1*	2,03,910	Tensin 1	.36	−.27

Note: (FC > 1.2 or <.83, *p* < .05; fold change = log_2_FC).

### 3.6 XST increases fatty acid metabolism in AMI rats by enhancing the expression of Echdc2, Gcdh, Dlst, and Nampt

Early acute myocardial injury leads to a disorder in carbon metabolism, which mainly manifests as fatty acid metabolism dysfunction. According to the results of proteomics analysis in Table 2, AMI, in 20 min, reduced 10 fatty acid metabolism proteins, which played an important role as enzymes. Interestingly, three enzymes reduced by injury were significantly reversed by XST; they were Echdc2, Gcdh, and Dlst, which was confirmed by Western blotting analysis (Figure 7C). The results showed that the abundance of Echdc2, Gcdh, and Dlst was inhibited after AMI in 20 min and was reversed by XST.

**TABLE 2:** DEPs of Sham: model enriched in fatty acid catabolism pathways.

Fatty acid catabolism pathway	DEP	Sham: model
Short-chain fatty acid catabolic process	Phyh and Acads	Down
Acyl-CoA biosynthetic process	Gcdh, Acat1, and Dld	Down
Fatty acid beta-oxidation	Gcdh, Acat1, Echdc2, Phyh, and Acads	Down
Acyl-CoA metabolic process	Dlst, Gcdh, Acat1, Dld, Dbi, and Suclg1	Down
Fatty acid metabolic process	Gcdh, Acat1, Echdc2, Phyh, Acads, Dld, and LOC679748	Down
	Prkaa2	Up

NAD is closely linked to fatty acid metabolism and the tricarboxylic acid (TCA) cycle, whereas Nampt participates in the NAD biosynthetic process. In this study, early acute myocardial injury elicited upregulation of Nampt (^#^ *p* < .05 vs. Sham), and the increase was apparently reversed by XST, which was confirmed by Western blotting analysis (Figure 7C).

### 3.7 XST alleviates AMI injury by attenuating the expression of Ywhaz and Ywhab

Here, 14-3-3 dimers act as the beginning of apoptosis by activating BAD and translocation to mitochondria. Ywhaz and Ywhab are members of the 14-3-3 protein family, which are also engaged in regulating a large spectrum of both general and specialized signaling pathways. Proteomics analysis showed that XST could lower the abundance of Ywhaz and Ywhab. Western blotting examination (Figure 7C) demonstrated that AMI induced elevation of Ywhaz (^##^ *p* < .01 vs. Sham) and Ywhab, and these alterations were reversed by XST treatment. This change was significantly exhibited in Ywhab (^**^ *p* < .05 vs. model).

### 3.8 Ywhaz might be a potential target of XST-alleviated AMI injury in accordance with molecular docking and SPR assay

**FIGURE 8:**
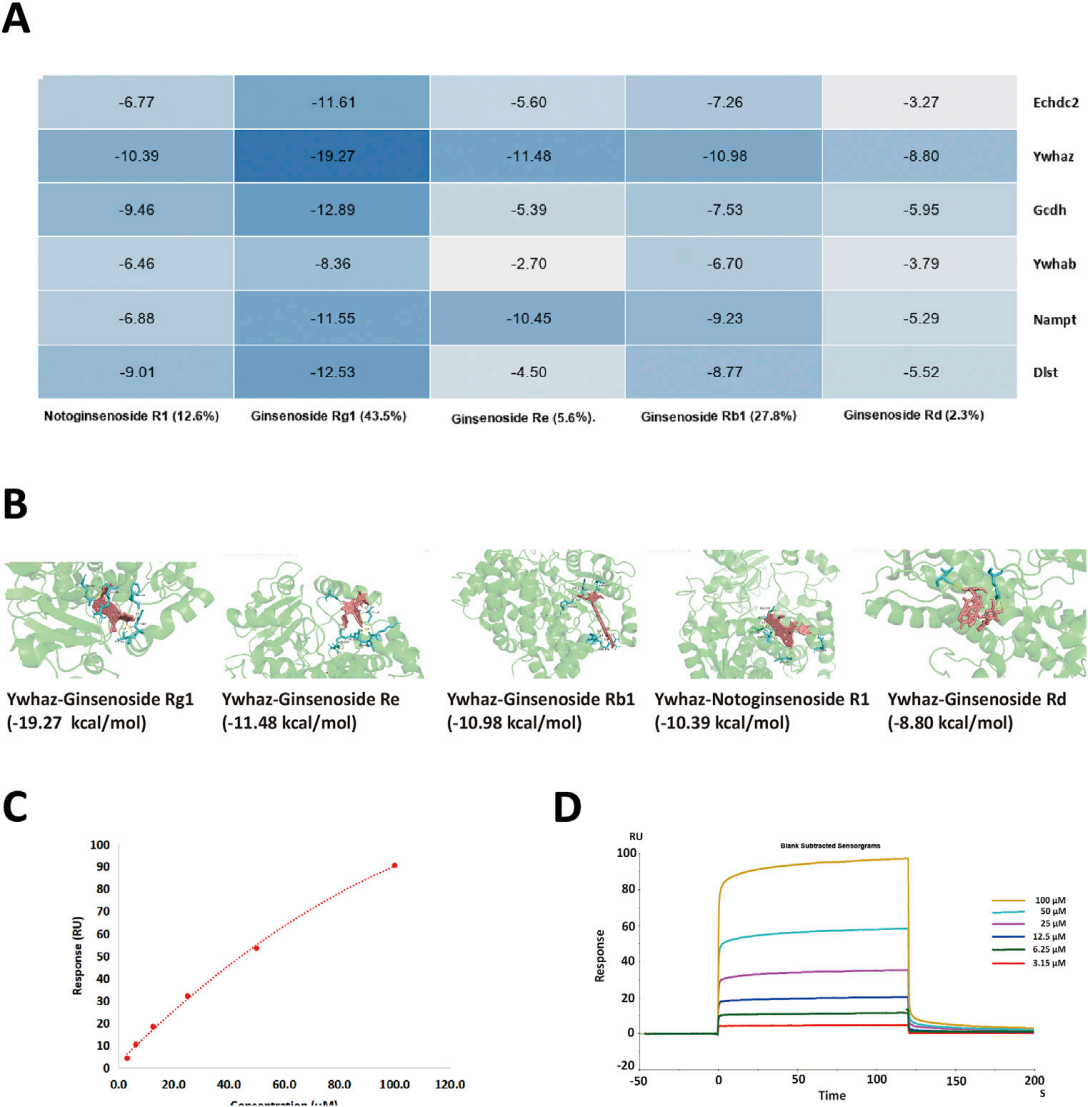
**(A)** Heatmap of binding energy between the five small molecule ligands and six DEPs, respectively. The darker the blue color, the lower the binding energy (kcal/mol) and the better the interaction are. **(B)** 3D structure of Ywhaz interactions. The key residues that contact the chemical compound are labeled in cyan. The chemical compound is shown in pink. **(C,D)** SPR assay analysis of the binding between Ywhaz and ginsenoside Rb1 at different concentrations. The open bar in **(D)** indicates the duration of ginsenoside Rb1 perfusion in SPR.

The six aforementioned core DEPs underwent molecular docking with the leading five ingredients of XST. The heatmap of docking scores is shown in Figure 8A. The more stable the binding between the ligand and the receptor, the lower is the binding energy of the two, and our results showed that the binding energy of the active ingredients and the four core proteins was ≤−7 kcal/mol. Furthermore, Ywhaz has a generally stable binding with all five compounds. The molecular docking diagrams are shown in Figure 8B.

To further verify whether the binding happened, the affinity measurement was tested between Ywhaz and the five ingredients. Among the result shown in Table 3, all five ingredients could directly bind to Ywhaz. Interestingly, the SPR assay showed that ginsenoside Rb1 binds with the highest affinity (1.576 μM) to Ywhaz (Figures 8C, D). These results suggested Ywhaz might be a potential target of XST-alleviated AMI injury, and ginsenoside Rb1, a key component of XST, might shoulder the drug effect on Ywhaz.

**TABLE 3:** K_D_ of the five ingredients binding to Ywhaz protein.

Ingredient name	K_D_ (μM)
Ginsenoside Rg1	399.8
Ginsenoside Re	44.86
Ginsenoside Rb1	1.576
Notoginsenoside R1	3,585
Ginsenoside Rd	240.5

## 4 Discussion

In this study, the AMI model was established by LAD ligation, and XST (38 mg/kg) was injected into the caudal vein 15 min before surgery. Within 20 min after the establishment of the AMI model, the pathological damage was evaluated, and the biological activity and mechanism of XST treatment were discussed. The results showed that the S-T segment, left ventricular function, arterial hemodynamics, and left ventricular hemodynamics were significantly different in the model group compared with the sham group. HR, SBP, DBP, LVSP, +*dp*/*dt*_max_, −*dp*/*dt*_max_, EF, FS, and SV all changed significantly. LVEDP and CO showed an obvious injury trend but did not reach a significant level, which may be related to the individual differences in early AMI injury in animals.

XST is an intravenous preparation of *P. notoginseng* saponins with more than 40 years of clinical experience. Current studies suggest that its anti-AMI mechanism is related to improving microcirculation disorders, regulation of coagulation function (Liu et al., 2020; Liu et al., 2021), and platelet aggregation function (Han et al., 2019). However, there are few in-depth molecular studies on the interaction mechanism of XST components against AMI. Diltiazem is a calcium antagonist that can inhibit the calcium overload, regulate calcium homeostasis, regulate myocardial and vascular cell functions, and antagonize AMI injury. It is used as an anti-myocardial infarction and anti-hypertension drug. Pathological results showed that XST could alleviate the early damage of AMI. These results suggest XST can reduce cardiac function injury in early AMI model rats, showing no more negligible protective effect than diltiazem.

To further investigate the cardio-protective mechanism of XST, we used the protein quantification method to evaluate and quantify the left ventricular total proteome concentration in the sham operation group, AMI model group, and XST (38 mg/kg)-treated group. The AMI group and XST group had seven significantly changed and overlapped DEPs, and six differentially expressed proteins (Echdc2, Ywhaz, Gcdh, Ywhab, Nampt, and Dlst) were detected in heart tissue. Western blot analysis verified that three protein expressions were upregulated and three were downregulated in the heart of rats in different treatment groups, which confirmed the results of quantitative protein detection. Echdc2, Gcdh, and Dlst are involved in fatty acid metabolism among these differentially expressed proteins.

Echdc2 is a mitochondrial protein, and previous studies proved that it could participate in regulating cell death and myocardial injury by increasing branched-chain amino acid metabolism (Du et al., 2013). Moreover, recent studies have shown that overexpression of Dlst plays an essential role in heart rate modulation by controlling ATP reduction in the heart, furthermore maintaining cardiac pacemaker cell function (Keßler et al., 2015; Heggermont et al., 2017). Gcdh acts as an oxidoreductase and participates in the acyl-CoA metabolic process (Goodman, 1981) and fatty acid beta-oxidation (Reichmann et al., 1988), which provides a possible link between Echdc2 and Dlst. The aforementioned proteins belong to fatty acid metabolism and appear to be regulators linking cell metabolism with cardiovascular disease. The reduction of the aforementioned fatty acid metabolism-related enzymes could be upregulated by XST treatment.

In addition to regulating metabolic pathways, DEPs found in this study may also affect other biological activities related to AMI injury. Ywhab and Ywhaz belong to 14-3-3 family proteins. They exist as dimers (homo- or hetero-dimer) in cells and may play a potential role in dimer/monomer dynamics and recently reported moonlighting chaperone-like activity (Sluchanko and Bustos, 2019). According to the analysis of the Reactome database, Ywhab and Ywhaz could involve in regulating a large spectrum of both general and specialized signaling pathways, including activation of BAD and translocation to mitochondria, regulation of localization of FOXO transcription factors, Rap1 signaling, and Chk1/Chk2(Cds1)-mediated inactivation of the cyclin B:Cdk1 complex. It is worth mentioning that Ywhaz was reported to reduce platelet phosphatidylserine (PS) exposure and procoagulant function to regulate arterial thrombosis (Schoenwaelder et al., 2016; Vilahur et al., 2018), which could match the regulation of coagulation function and platelet aggregation function of XST. In this study, the expression of Ywhab and Ywhaz were upregulated in early AMI stimulation, suggesting early AMI may lead to abnormal platelet procoagulant function and XST can reverse these changes.

With the deepening of mechanism research, the interplay between ginsenosides and the aforementioned proteins has been increasingly focused upon (Chen et al., 2021; Zhu et al., 2021). To further explore the action mechanism and target of XST active ingredients, we used molecular docking technology to predict the binding energy between the five main components of XST and key DEPs. Our molecular results showed that the binding energies of over half interactions were ≤−7 kcal/mol, indicating that the aforementioned active ingredients had good binding for their target. Among them, Ywhaz has a generally stable binding with all five compounds of XST. To verify the interaction between Ywhaz and the five compounds of XST, SPR assay was applied to measure the affinity of the interactions. Similar to molecular docking, Ywhaz could directly bind with all five compounds. Moreover, Chen and his colleagues proved that the Ywhaz protein is a potential target of ginsenosides through crystallographic and mutagenesis methods (Chen et al., 2021). This indicates that Ywhaz may be the core target of XST when treating AMI in the early stage.

## 5 Conclusion

Our study demonstrates that cardiac function in rat models was impaired within 20 min of the onset of AMI. XST prophylaxis has a particular ameliorative effect. Proteomic analysis of heart tissue was detected using a label-free technology. A total of 117 essential DEPs were identified, including 80 in Sham: model and 53 in Model: XST. Intensive bioinformatics analysis identified that fatty acid metabolism and platelet procoagulant function were mainly involved. Furthermore, Western blot assay verified that Echdc2, Ywhaz, Gcdh, Ywhab, Nampt, and Dlst play an essential role in the protective effect of XST. Finally, molecular docking analysis and SPR assay investigated that Ywhaz might be the core target of XST when treating AMI in the early stage. Overall, this study has highlighted some of the multiple components of Xueshuantong injection and provides evidence for several potential therapeutic targets, which may be involved in its beneficial effects on AMI in the early stage.

## Data Availability

The datasets presented in this study can be found in online repositories: Proteomics data are available *via* the ProteomeXchange Consortium with the identifier PXD035370. Raw data and figures are available on Nutstore (https://www.jianguoyun.com/c/sd/158c9e6/334b295c5999971b).
